# High Pressure Phase-Transformation Induced Texture Evolution and Strengthening in Zirconium Metal: Experiment and Modeling

**DOI:** 10.1038/srep12552

**Published:** 2015-07-28

**Authors:** Xiaohui Yu, Ruifeng Zhang, David Weldon, Sven C. Vogel, Jianzhong Zhang, Donald W. Brown, Yanbin Wang, Helmut M. Reiche, Shanmin Wang, Shiyu Du, Changqing Jin, Yusheng Zhao

**Affiliations:** 1 Beijing National Laboratory for Condensed Matter Physics and Institute of Physics, Chinese Academy of Sciences, Beijing 100190, China; 2 Materials Science and Technology Division, Los Alamos National Laboratory, Los Alamos, New Mexico, 87545, USA; 3 School of Materials Science and Engineering, Beihang University, Beijing 100191, China; 4 Center for Advanced Radiation Sources, The University of Chicago, Chicago, Illinois, 60469, USA; 5 HiPSEC and Department of Physics and Astronomy, University of Nevada, Las Vegas, Nevada, USA; 6 Division of Functional Materials and Nanodevices, Ningbo Institute of Materials Technology and Engineering, Chinese Academy of Sciences, Ningbo, Zhejiang 315201, China; 7 Collaborative Innovation Center of Quantum Matter, Beijing, China

## Abstract

We studied the phase-transition induced texture changes and strengthening mechanism for zirconium metal under quasi-hydrostatic compression and uni-axial deformation under confined high pressure using the deformation-DIA (D-DIA) apparatus. It is shown that the experimentally obtained texture for ω-phase Zr can be qualitatively described by combining a subset of orientation variants previously proposed in two different models. The determined flow stress for the high-pressure ω-phase is 0.5–1.2 GPa, more than three times higher than that of the α-phase. Using first-principles calculations, we investigated the mechanical and electronic properties of the two Zr polymorphs. We find that the observed strengthening can be attributed to the relatively strong directional bonding in the ω phase, which significantly increases its shear plastic resistance over the α-phase Zr. The present findings provide an alternate route for Zr metal strengthening by high-pressure phase transformation.

Zirconium is a fascinating 4d transition metal widely used in nuclear, chemical, and biomedical industries due to its small neutron absorption cross section and high resistance to corrosion. At ambient conditions, zirconium crystallizes in a hexagonal close packed (hcp) structure known as the α phase. Above 1135 K at atmospheric pressure, it transforms to a body-centered-cubic structure, commonly referred to as the β phase. With increasing pressure, α-Zr (P6_3_/mmc) transforms into another hexagonal structure, called the ω phase (P6/mmm), which is not close packed and has three atoms per unit cell^1^,^2^,^3^. The ω-Zr can be recovered as a metastable phase at ambient conditions when pressure is released^4^,^5^,^6^, but it will transform back to the α phase at elevated temperatures above 470 K^7^.

The ω structure has also been observed in various zirconium-based alloys^8^,^9^, an interesting phenomenon that has received extensive experimental and theoretical attention. Although widely investigated, the atomic-scale mechanisms underlying the α–ω phase transformation in Zr are still controversial and not fully understood. Based on transmission electron microscopy (TEM) on samples recovered from high pressure, two different orientation relationships between the α and ω phases of Zr have previously been reported. The first, 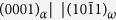 and 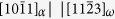 , proposed by Usikov and Zilbershtein (hereafter referred to as UZ) involves atoms that form the basal plane in an α unit cell being shuffled into a pyramidal plane of the ω unit cell^4^. The second, 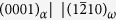 and 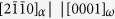, observed by Botstein *et al.* (hereafter referred to as BRT), proposes that atoms within the basal plane of an α unit cell are re-arranged to form a prismatic plane of the ω cell^5^. This relation was further confirmed recently by the texture analysis of Wenk *et al.*^10^.

The α-ω phase transformation in Zr is of martensitic type. As the result of lattice reconstruction, the atomic packing in the ω phase is 2.4% more efficient than in the α phase, which could in principle lead to improved mechanical properties^1^. Indeed, based on nano-indentation measurements^6^, the ω-phase Zr recovered from high pressure shows an 80% increase in hardness over the α-Zr. Zhao and Zhang also reported strengthening of ω-phase Zr from the diffraction peak-width analysis of microscopic strain^11^. These findings suggest that pressure-induced phase transition leading to meta-stable phases at ambient condition is an effective route for material strengthening. However, the constitutive law and related deformation mechanism for ω- Zr have yet to be established in its stability field. In this work, we investigate the α-ω phase transformation mechanism by analyzing the texture pole figure features and determine the strain-lattice stress relation for ω-phase Zr in a deformation-Diamond (D-DIA) apparatus. Furthermore, the strengthening mechanism of ω phase Zr is revealed by first-principles calculations.

## Results

### Texture evolution Analysis

**Figure 1:**
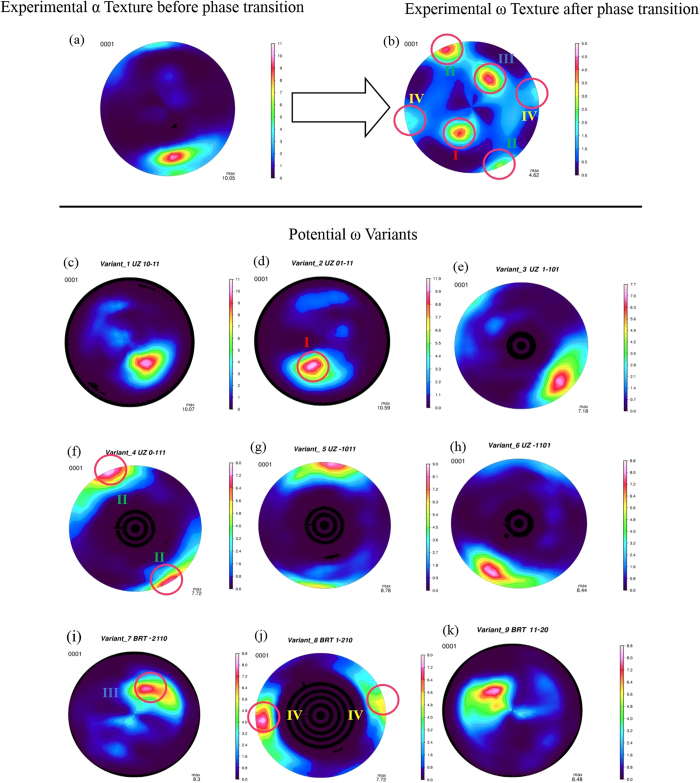
The c-axis (0001) pole figures before the phase transformation (**a**) and after a complete transformation to ω-Zr at 5.9 GPa (**b**). (**c**–**h**) are pole figures obtained for ω-Zr when applying each of the six different variants in the UZ model to the initial alpha texture. (**i**–**k**) show the three different ω phase pole figures predicted by the BRT model.

The texture information of Zr before (α phase) and after phase transformation (ω phase) is analyzed by Rietveld method using the “Material Analysis Using Diffraction (MAUD)”^12^. For the α phase, our MAUD refinement reveals a strong initial basal fiber texture in which the fiber axis is vertical, although slightly tilted, to the plane of the (0001) pole figure, as illustrated in Fig. 1(a). This is consistent with previous texture analysis and is also expected for a clock-rolled material. The observed tilt in the fiber axis indicates that the sample is slightly misaligned during sample mounting and initial compression. After the transformation, the ω phase shows a different (0001) pole figure for the c-axis from the α phase. The (0001) pole figure of the ω phase is comprised of four distinct maxima, labeled as I to IV in Fig. 1(b), allowing us to determine the variant selection.

Due to the symmetry of hexagonal structures and the orientation relations, each orientation has several symmetry-equivalent variants. ODFvf3 program (Tomé, C.N. ODFVF3, Version 14th (2008), Los Alamos National Lab, USA.) can read texture files and possible orientation relationships between the α and ω phases and then generates an output texture for each variant, i.e. compute the phase transformation texture as if only a single of the equivalent variants would be active. For the α phase, by simply applying an individual variant to the initial experimental texture, we can use the ODFvf3 program to derive the contributions of all possible variants to the pole figure after the phase transition. In the UZ relationship, 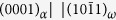 and 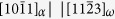, all six variants for the ω phase are unique, corresponding to six different pole figures, Fig. 1(c–h). By contrast, in the BRT relationship, 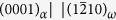
and 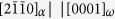 , generates only three unique variants 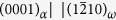 and 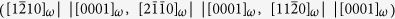, as shown in Fig. 1(i–k), which are also different from the variants calculated for the UZ orientations relationship. Further inspection of Fig. 1 shows that the experimentally obtained texture for the ω phase is an overlay of a subset of these nine different variants (d, f from UZ model and i, j from BRT model), indicating that both UZ and BRT orientation relationship are required to explain the observed omega texture. This pole figure analysis therefore demonstrates that both the UZ and the BRT orientation relationships are active for the high-pressure α to ω-Zr phase transition.

### Lattice-plane dependent flow-curves derived from diffraction patterns

**Figure 2:**
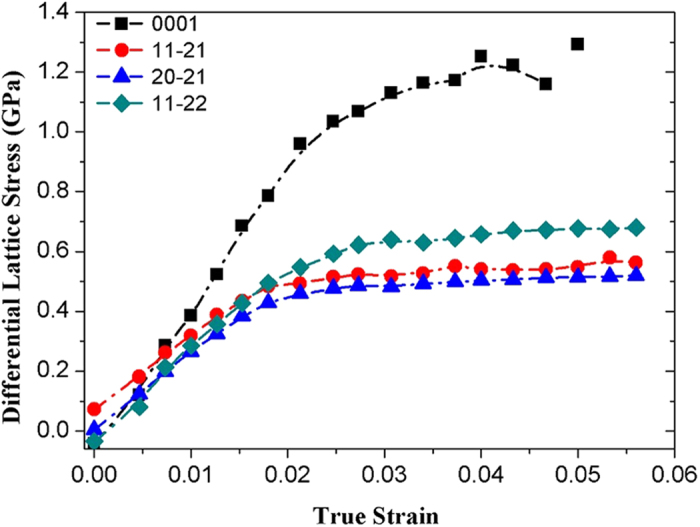
The macroscopic strain-lattice stress curves derived for different lattice planes of the ω-phase Zr during compressive deformation. The data is terminated at 0.05 total strain for the (0001) plane because its diffraction signal become scattered at higher strains. The deviation of data points from zero at zero strain can be taken as uncertainties in the lattice stress calculations.

After the phase transition, the lattice-plane dependent flow-curves for the ω phase Zr, i.e. flow-curves with the strain derived from the macroscopic length change of the sample and the lattice stresses calculated from observed lattice strains (peak-shifts) and single crystal elastic constants, are derived by using Multifit and Polydefix software developed by Sebasian Merkel (available at http://merkel.zoneo.net/Multifit/). The flow-curves for different lattice planes, shown in Fig. 2, are derived from the (0001), (11–21), (20–21), (11–22) peaks. Below 0.017 total strain, the ω-phase Zr deforms elastically, which corresponds to the linear region of the stress-strain curve. At higher strains, all stress-strain curves start to deviate from the linearity, indicating that the ω-Zr starts to yield and deform plastically. In Fig. 2, we observe that the (20–21) peak is the most compliant plane while the (11–21) is only slightly stronger than the (20–21) plane. Not surprisingly, they exhibit a similar flow stress of ~0.5 GPa at total strain of 0.06. Compared with (11–21) and (20–21), the (11–22) plane is much stronger, and the determined stress is 0.68 GPa at 0.06 strain. The (0001) is the least compliant plane and shows an extraordinary strength during the deformation. The determined ultimate flow stress for the (0001) plane is 1.2 GPa at 0.04 strain, which is more than twice higher than those of other lattice planes.

Strikingly, the overall flow strength of the ω-phase Zr (0.5–1.2 GPa) is substantially higher than that of the α phase (0.1–0.2 GPa)^11^, indicating that pressure-induced phase transition is an alternate route for material strengthening. With conventional strengthening techniques such as strain hardening, filming or alloying, it can further be expected that the ω-Zr may exhibit even better mechanical performance. In addition, alloying or filming is well adopted by industry and could be more practical in the real applications. The enhanced resistance to plastic strain in the ω-Zr could be due to the localization of the d-electrons and the formation of directional bonding after the high-pressure phase transition. Presumably, the directional bonding should be in the c direction given the extraordinary strength for the (0001) lattice plane. More detail strengthening mechanism will be discussed in the subsequent paragraphs.

### Strengthening mechanism investigated by First-principles calculations

We now turn to discuss the relative ductility or brittleness for the two structures of zirconium in a qualitative manner. The elastic constants were determined from the first principles calculations by means of efficient strain energy methods as described in Ref. 13,14. The calculated elastic constants, bulk modulus and shear modulus of α- and ω-phase Zr are listed in Table 1. Whether a crystalline solid is intrinsically ductile or brittle can be approximately determined by the ratio (specific to the solid) of the shear modulus (G) to the bulk modulus (B), i.e. G/B, by simply considering B as the resistance to fracture and G the resistance to plastic deformation^15^,^16^. The critical G/B ratio that separates ductile and brittle materials is around 0.57, G/B < 0.57 for a ductile behavior and G/B > 0.57 for a brittle behavior. While both phases of Zr are ductile according to our first principles calculations (Table 1), the α-Zr has a relatively lower G/B ratio (~0.36) than the ω-Zr (0.46), suggesting that the ω-Zr has higher plastic resistance due to its stronger directional bonding, which will be further clarified by comparison of their electronic structures below. Note that a more advanced brittle-ductile criterion proposed by Kelly *et al.*^17^ is not considered here and may be used for further studies.

**Figure 3:**
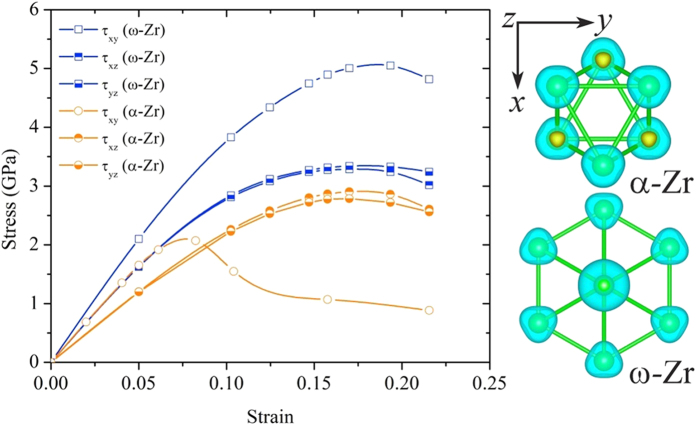
The calculated stress-strain relationships for the α- and ω-Zr under shear deformations.

The anisotropic ideal strength is another important intrinsic mechanical parameter for defining the upper limit of the strength of a real crystal and also for governing the dislocation nucleation in a defect-free crystal^18^. On the other hand, the materials’ strength or hardness in a real material is governed by various defects. In this regard, the ideal shear strength can be factored into the dislocation-mediated plastic resistance by controlling the dislocation core width through the Peierls-Nabarro stress model^19^,^20^. As demonstrated in this model, the introduction of ideal shear strength is capable of capturing some of the large differences in chemical bonding, which cannot be revealed by elastic constants^21^. The ideal shear strength can also provide the bond resistance to large and irreversible shear strains within the lattice/electronic stability, at which the lattice/electronic instability occurs, while elastic constants can only determine the lattice tolerance around equilibrium or associated with reversible lattice distortion. To account for the effect of ideal shear strength on plastic resistance, the strain-stress relationships are calculated for the two Zr polymorphs, in a similar manner to our previous studies of other materials^22^. Figure 3 shows the calculated strain-stress curves for the α and ω-Zr under various shear deformations modes. The maximum stress in the curve corresponds to the ideal strength along a specific deformation path, after which the lattice/electronic instability occurs. Interestingly, we find that the weakest deformation path is along {11–20}<10-10> for α-Zr, in agreement with the results of deformation experiments^23^. The weakness on {11–20}<10-10> is also responsible for the dislocation mobility in this slip system for α-Zr and the observed texture evolution. In addition, the calculated minimum shear strength for α-Zr (~2.1 GPa) is about 36% lower than that of ω-Zr (~3.3 GPa) (Fig. 3), which agrees with our experimental results, suggesting that ω-Zr is intrinsically stronger than α-Zr. For both phases, the shear strength is isotropic within the basal planes due to their hexagonal symmetry. However, the weakest deformation path changes from {11–20}<10-10> shearing in α-Zr to the basal-plane shearing mode in ω-Zr, suggesting a difference in the preferred slip system for the two Zr structures.

To underline more realistically the plastic resistance of a real material, a simple but not trivial model is based on the qualitative analysis of model parameters in the Peierls-Nabarro stress model, which is generally expressed as 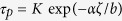, where *α* = 2π and *K* = G/(1-ν) for edge dislocation, *K* = G for screw dislocation. G is the shear modulus and ν the Poisson ratio, *ζ* is the dislocation half-width, which can be expressed as *ζ* = *Kb*/4*πτ*_max_. It is clear that the plastic resistance of a crystal depends mostly on two intrinsic mechanical parameters: shear modulus G and ideal shear strength *τ*_max_. For a more physical interpretation *τ*_max_ can be viewed as the maximum slope of the generalized stacking fault energy profile or simply as the ideal shear strength under well-defined deformation mode along the weakest link that promotes the mobility of dislocations. Based on the calculated elastic constants (Table 1) and minimum shear strengths: G_v_ = 35.4 GPa and *τ*_min_ = 2.1 GPa for α-Zr and G_v_ = 44.7 GPa and *τ*_min_ = 3.3 GPa for ω-Zr, it is found that ω-Zr possesses a ~26% higher shear modulus and ~60% higher ideal shear strength than the corresponding values of α-Zr, further suggesting higher plastic resistance for the ω-Zr. These results are in agreement with our experimental results that ω-Zr exhibits a significantly higher yield strength than α-Zr, indicating a consistency between experiments and theoretical analysis.

**Figure 4:**
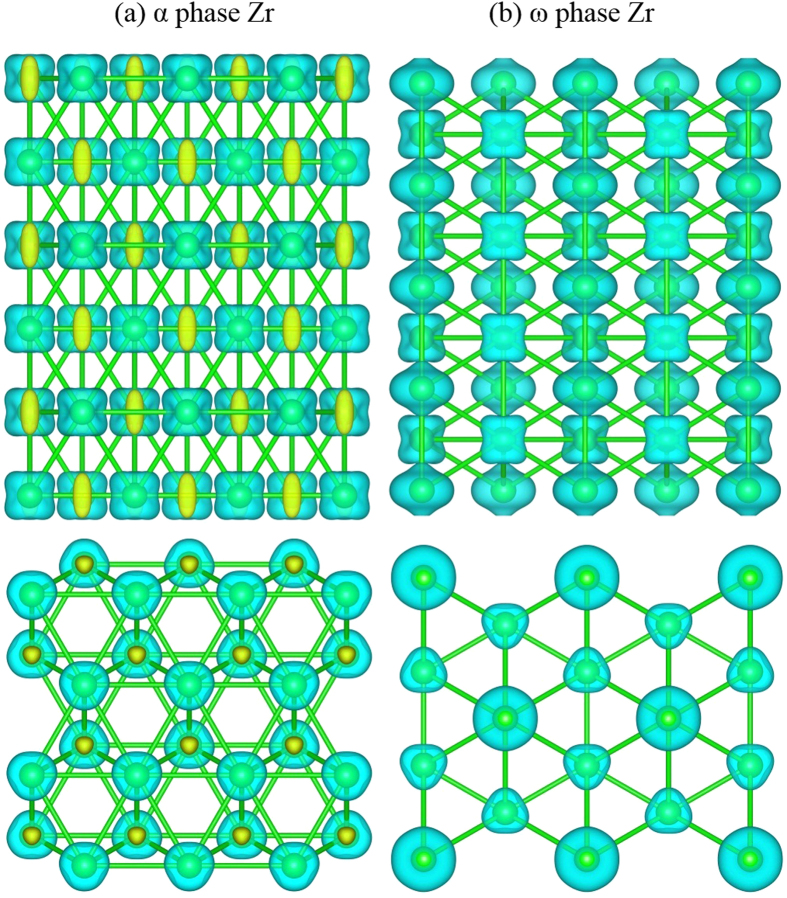
The isosurface maps of valence charge density difference (VCDD) for (**a**) α-Zr and (**b**) ω-Zr. The isosurfaces of VCDD correspond to +/−0.01 electrons/Bohr^3^. The green and orange colored isosurfaces correspond to negative and positive values, respectively.

**Figure 5:**
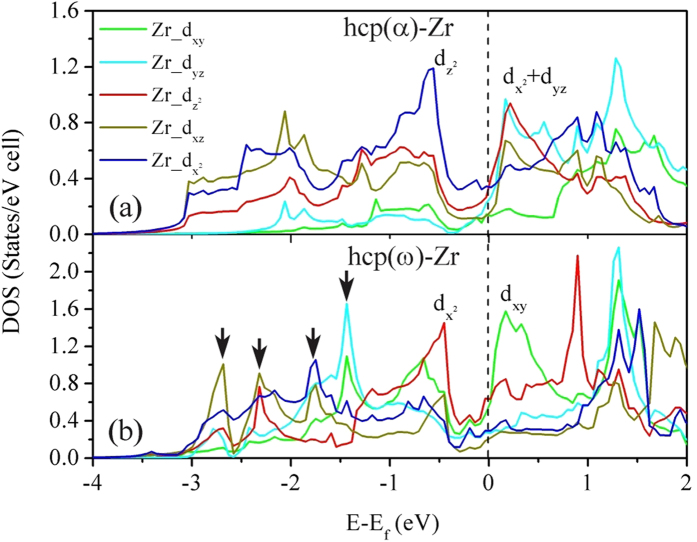
Orbital decomposed partial electronic density of state of α-Zr and ω-Zr. The vertical dashed lines indicate the Fermi levels.

In order to gain deeper understanding into the plastic behavior for the two polymorphs of Zr, the valence charge density differences (VCDD) are calculated and compared in Fig. 4. It is seen that the charge depletion at the center of hexagons in the α structure is markedly different from that in the ω-Zr. In addition, the ω-Zr has a distinct and homogeneous distribution of positive VCDD, suggesting a higher polarization. To account for the electronic origin of such differences, the orbital decomposed electronic density of states (DOS) are calculated and shown in Fig. 5. It is seen that both Zr phases show finite DOS at the Fermi level and hence exhibit metallic behavior. However, the ω-Zr shows strong localization of d orbitals as revealed in Fig. 5 (i.e., peaks indicated by the arrows), while the delocalization contribution can be reflected from the smoother DOS profile for the α-Zr. These calculations indicate clearly a stronger component of directional bonding for the ω phase. Furthermore, the strong directional bonding character for the α-Zr is mostly attributed to the hybridization of dz^2^ orbitals, while the bonding in the ω-Zr is from hybridization of dx^2^ orbitals (see the peak just below the Fermi level in Fig. 5). It is known that the directional bonding or covalency can significantly lower the intrinsic resistance to dislocation mobility as compared to metallic bond character, because the local chemical structure must change around the dislocation cores in covalent materials, which would in turn result in a larger barrier to dislocation motion. Another important feature of the DOS in Fig. 5 is the presence of the so-called “pseudo-gap” for both Zr phases, which is the difference between the bonding and anti-bonding states. This suggests that a strong “pseudo-covalent” contribution exists in both structures although it is stronger for the ω-Zr. All these differences in chemical bonding further support our earlier argument that the relatively strong directional bonding provides an electronic origin of a higher plastic resistance for the ω-phase Zr. Our first principles simulation results are consistent with the augment by Jamieson^1^, who suggested that one of the valence electrons in the ω-Zr may adopt a strictly localized d-state for each atom, hence leaving only three electrons behind to form the conduction band.

## Discussions

The atomic-scale mechanisms underlying the α-to-ω phase transformation in Zr are revealed by analyzing the pole figure configuration. It is shown that both the UZ model-{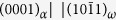 and 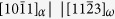} and the BRT model-{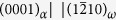 and 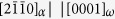} are active for the orientation relationship during the α-to-ω phase transition. After the phase transition, the ω-phase was deformed in a compressive mode to derive the constitutive law for the ω-phase Zr. The determined flow stress is 0.5–1.2 GPa, more than three times higher than that of α-phase Zr at 6 GPa. Based on first-principles calculations of electronic structure, the observed strengthening and associated high resistance to plastic deformation can be attributed to the relatively strong directional bonding in the ω phase. The present findings confirm that the high-pressure phase transition can be an effective route for Zr metal strengthening.

## Methods

The sample from Alfa Aesar is a clock-rolled cylinder of 1.2 mm diameter and 1.2 mm length. The experiment was performed at GSECARS beamline 13-BM-D at the Advanced Photon Source (APS) synchrotron, Argonne National Lab by using a deformation-diamond (D-DIA) apparatus^24^. The D-DIA apparatus is a modified version of DIA that uses 6 hard anvils (primarily WC) to compress a sample assembly. For deformation under confined high pressure, the two vertical anvils can be driven independently to introduce shortening or lengthening deformation. The incident monochromatic X-rays of 65 keV were collimated by WC slits to 200 × 200 μm for diffraction. By taking the slits out of the beam path, radiographic images of the cell assembly during the deformation were collected to measure the sample length. With X-ray transparent anvils made of cubic boron nitride, the entire Debye-Scherrer rings were recorded by a two dimensional CCD detector for texture analysis and lattice strain measurements.

The sample was first compressed hydrostatically at room temperature to 6 GPa until the α phase was completely transformed to ω phase. At this confining pressure, the uniaxial deformation up to 0.06 strain was achieved by advancing two vertical differential rams at a constant strain rate of 10^−5^/s. The methods used here for macroscopic stress analysis are similar to those described by ref. 25, 26, 27. We first determine lattice strain, *ε*(*φ*) = [d_0_(*φ*)−d(*φ*)]/d_0_(*φ*), where *φ* is the true azimuth angle, given by cos*φ* = cos*θ*cos*χ* (θ and χ are the diffraction angle and detector azimuth), and d_0_(*φ*) and d(*φ*) are d-spacing values of a given lattice plane at the onset of deformation and at a certain stress state, respectively. The obtained lattice strain, *ε*(*φ*), is then fitted to the following equation to determine the differential lattice strain, *ε*_*t*_.


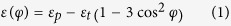


With a cylindrical symmetry of the stress field in the D-DIA pressure cell the differential stress is defined by *t* = *σ*_1_−*σ*_3_, where *σ*_1_ and *σ*_3_ are the principal stresses in axial and radial directions, respectively. For a given lattice plane *hkl*, the differential strain can be converted into the differential stress by


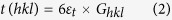


where *G*_*hkl*_ is the shear modulus for the lattice plane *hkl*.

The sample columns from X-ray radiographic images are used to measure the changes in the sample length during the deformation^28^. Depending on the intensity contrast between sample and strain marker (Au foils), the sample length changes can be measured within 1 micron resolution. The total axial strain of the sample is calculated by: ε = (*l*-*l*_0_)/*l*_0_, where *l*_0_ is the sample length at the onset of deformation and *l* is the sample length under a given stress condition.

First-principles calculations were performed using the VASP code within the generalized-gradient approximation proposed by Perdew and Wang for exchange-correlation functional^29^,^30^. The integration in the Brillouin zone was done on special k points for the crystalline phases under consideration, which are determined according to the Monkhorst-Pack scheme. The tetrahedron method with Blöchl corrections was used for the energy and electronic calculations at the energy cutoff of 600 eV, and the Methfessel-Paxton smearing scheme is adopted for the stress/force relaxations. The conjugate gradient method was used for the relaxation of structural parameters. To check the reliability of our calculations, the structural and elastic properties for both α- and ω-Zr are calculated and then compared with available theoretical and experimental values. These calculations provide a unified set of elastic constants/moduli for α- and ω-Zr, which are used for further investigation of some relevant mechanical properties such as ductility or brittleness and Peierls-Nabarro stress analysis.

## Additional Information

**How to cite this article**: Yu, X. *et al.* High Pressure Phase-Transformation Induced Texture Evolution and Strengthening in Zirconium Metal: Experiment and Modeling. *Sci. Rep.* **5**, 12552; doi: 10.1038/srep12552 (2015).

## Figures and Tables

**Table 1:** Elastic constants, bulk modulus and shear modulus of α- and ω-phase Zr calculated from first-principles calculations.

	C11	C12	C13	C33	C44	B	G
α	148.5	66.9	70.5	165.7	25.7	97.6	35.4
ω	160.9	73.7	53.6	195.1	33.8	97.4	44.7

All units are in GPa. The results are in good agreement with ref 31.
